# The Clinical Sustainability Assessment Tool: measuring organizational capacity to promote sustainability in healthcare

**DOI:** 10.1186/s43058-021-00181-2

**Published:** 2021-07-17

**Authors:** Sara Malone, Kim Prewitt, Rachel Hackett, John C. Lin, Virginia McKay, Callie Walsh-Bailey, Douglas A. Luke

**Affiliations:** 1 Washington University in St. Louis, Brown School, St. Louis, MO USA; 2 Division of Pediatric Infectious Disease, Department of Pediatrics, Washington University School of Medicine, St. Louis, USA; 3 Division of Pediatric Critical Care Medicine, Department of Pediatrics, Washington University School of Medicine, St. Louis, USA

**Keywords:** Sustainability, Healthcare, Clinical science, Measurement development, Reliability

## Abstract

**Background:**

Few validated assessment tools are available to increase understanding and measure factors associated with sustainment of clinical practices, an increasingly recognized need among clinicians. We describe the development of the Clinical Sustainability Assessment Tool (CSAT), designed to assess factors that contribute to sustainable practices in clinical settings.

**Methods:**

Sixty-four participants from clinical and research fields participated in concept mapping and were recruited to brainstorm factors that lead to sustained clinical practices. Once repeated factors were removed, participants sorted items based on similarity and rated them by importance and feasibility. Using concept mapping analyses, items were grouped into meaningful domains to develop an initial tool. We then recruited pilot sites and early adopters, for a total of 286 practicing clinicians, to pilot and evaluate the tool. Individuals were recruited from clinical settings across pediatric and adult medical and surgical subspecialties. The data were analyzed using confirmatory factor analysis (CFA) to test hypothesized subscale structure in the instrument. We used root mean square error of approximation (RMSEA) and the standardized root mean square residual (SRMR) to assess fit and thus the ability of CSAT to measure the identified domains.

**Results:**

The concept mapping produced sorted statements that were edited into items that could be responded to, resulting in the creation of a tool with seven determinant domains and 47 items. The pilot and CFA testing resulted in a final CSAT instrument made up 35 items, five per domain. CFA results demonstrated very good fit of the seven domain structure of the CSAT (RMSEA = 0.049; SRMR = 0.049). Usability testing indicated the CSAT is brief, easy to use, easy to learn, and does not require extensive training. Additionally, the measure scored highly (18/20) on the Psychometric and Pragmatic Evidence Rating Scale (PAPERS). The seven final CSAT domains were engaged staff and leadership, engaged stakeholders, organizational readiness, workflow integration, implementation and training, monitoring and evaluation, and outcomes and effectiveness.

**Conclusions:**

The CSAT is a new reliable assessment tool which allows for greater practical and scientific understanding of contextual factors that enable sustainable clinical practices over time.

**Supplementary Information:**

The online version contains supplementary material available at 10.1186/s43058-021-00181-2.

Contributions to the literature

- Sustainability of evidence-based practices is important to deliver intended health outcomes to individuals and populations. However, there has been relatively little focus on sustainability theory and methods in implementation science.
- We introduce the Clinical Sustainability Assessment Tool (CSAT), a new instrument for measuring sustainability capacity in clinical and healthcare settings.
- The CSAT is designed to be easy to use—it is short, requires little training, and can be used by researchers, evaluators, and frontline clinical staff.
- The CSAT is one of the few reliable assessment tools for measuring sustainability and is the only tool that allows quick assessment of clinical sustainability by evaluators and quality improvement staff, as well as researchers.
- The CSAT has excellent usability and reliability, and preliminary validation data suggest that the CSAT is able to distinguish among different types of clinical settings.

## Background

Sustainability is the degree to which an evidence-based program, policy, or intervention can deliver its intended benefits over an extended period of time [1]. Programs need to be sustained and clinical interventions continuously delivered to achieve their desired public health impacts and clinical outcomes [2]. Sustainability can be challenging, and in fact, many initially implemented programs fail to remain in place over time. Evidence suggests that interventions that are not sustained could result in decreased care quality and worsened patient outcomes and quality of life [3, 4]. Programs that are not kept up after an initial investment can waste money and non-monetary resources [5, 6]. Having a better understanding of what factors affect sustainability can inform strategies to improve the likelihood that interventions will successfully continue after initial implementation [7, 8].

Studies within implementation science often focus on initial program or intervention adoption and implementation, placing less emphasis on sustainability [9]. Recent reviews of sustainability found relatively few studies focusing on sustainability, most of which were of limited quality and methodological rigor [4, 10, 11]. Research on sustainability is limited in part because data collection for grant-funded studies is typically not carried out long enough to determine predictors of long-term sustainment [12]. Consequently, there have been recent calls for increased attention to sustainability research, theory, and methods [4, 11]. In response to this, the Sustainment Measurement System Scale (SMSS) was developed to understand both determinants and outcomes of sustainment for mental health and substance abuse programs [8].

Partly to address this critical gap in the field, we previously developed the Program Sustainability Assessment Tool (PSAT), which was designed to measure capacity for program sustainability [13], particularly for evidence-based public health programs. The PSAT has been used worldwide for local, regional, and national programming [14]. Although it was developed for public health programs, it has also been used by social service, clinical care, and education programs to assess the sustainability of a variety of programs focused on transitional services, obesity prevention, behavioral health, health systems strengthening, and tobacco control [15].

The PSAT has been used to examine sustainability within clinical and healthcare settings, but early reports from users highlighted a number of limitations. Most essentially, clinical settings differ from public health settings in a number of important ways which change how sustainability should be conceptualized and measured. For example, funding in clinical settings tends to be tied primarily to public and private insurance funding, rather than public grants and/or private donations for public health initiatives [16]. Thus, the sustainability of a clinical practice is more tied to its ability to be reimbursed by payors [17, 18]. Furthermore, community outreach, a key driver of sustainability in public health settings, is conducted less in clinical settings as services are mainly delivered to identified in-services patients [19]. Current evidence suggests that sustaining evidence-based practices is particularly challenging in clinical settings. For example, a review of practices in the UK National Health Service (NHS) found that 33% of quality improvement projects are not sustained for 1 year after initial implementation [20, 21]. This speaks to the need for assessment procedures that are specially designed to assess the specific aspects of sustainability within healthcare and clinical settings.

Recently, a changing context in clinical healthcare has also become relevant to the implementation and integration of evidence-based practices. Clinical healthcare has widely adopted electronic medical records, dramatically changing how practitioners chart, build workflows, and communicate with other team members. This change has altered the structure in different health settings and how professionals orient themselves to medical decisions as well as other team members. New evidence-based clinical practices need to be integrated into this complex information and work system to be able to sustain them over time. Furthermore, sustainability outcomes in clinical settings play out over shorter time periods than for public health programming that is focused more on population health outcomes. Finally, the rapid cycle improvement that has historically been promoted in clinical settings and quality improvement initiatives has failed to focus on or understand the role of long-term sustainability and organizational capacity within this realm [22–24].

To address this need for additional research focused on sustainability and to support clinicians interested in planning for sustainable implementation of innovations, we present a new instrument for measuring sustainability capacity in clinical and healthcare settings: the *Clinical Sustainability Assessment Tool* (CSAT). In this paper, we describe the conceptual and measurement development of the CSAT, and present pilot data demonstrating its usability, reliability, and preliminary validation with a variety of healthcare practitioners and researchers. We also outline the next steps for the CSAT, including broader dissemination and validation of the tool.

## Methods

This is a measurement development study of a tool to assess capacity for sustainability in clinical healthcare settings. The study included two major components: (1) conceptual development guided by a literature review and expert opinion (via concept mapping), and (2) a pilot study of an initial version of the CSAT to assess its reliability, confirm its domain structure, and produce a final version of the instrument ready for dissemination and use in the field. The CSAT was designed to be easy to use by researchers, evaluators, and clinicians, and applicable to a wide variety of healthcare settings.

### Conceptual development

A literature search was conducted to identify any existing tools, surveys, instruments designed for assessing clinical sustainability. Two project team members (KP, SM) searched for articles about measures related to the sustainability of a clinical care or behavioral health practice. The search included journals within the fields of clinical medicine and implementation science as well as Scopus [25]. The article reference lists were also examined for relevant publications. The literature search results at the time showed that there was no simple, valid, and reliable tool for measuring the sustainability of a clinical care practice.

After the literature review, a concept mapping approach was conducted to develop the CSAT [26, 27]. Concept mapping is a mixed methods approach to reveal the structure of a complex conceptual domain and is a useful tool for measurement development [28, 29]. Concept mapping analysis and results were conducted using The Concept System® Global MAX™ software: Concept Systems, Inc. Copyright 2004-2021; all rights reserved [27].

We used concept mapping to conceptualize sustainability of practices in clinical settings, which resulted in the production and refinement of the domain structure for the measure. Concept mapping consisted of five steps, including (1) brainstorming, (2) sorting, (3) rating, (4) analysis of structure, and (5) cleaning/measure creation. Sixty-two participants from multiple professions and different healthcare settings participated in an initial brainstorming activity.

Concept mapping respondents included:

- Researchers in areas of clinical care, sustainability, and dissemination and implementation science (n=10)
- Clinical care administrators (e.g., nurse managers, medical directors, quality and safety leads; n=9)
- Clinical care service providers (e.g., physicians, nurses, rehabilitation specialists; n=43)

Participants represented various specialties and clinical environments (e.g. inpatient, outpatient, adult, and pediatric settings). In the brainstorming phase, these participants responded to the prompt, “In order for clinical practices to continue over time, they need…” and generated an initial list of 230 statements. The study team (DL, KP, SM, RH, and JL) reviewed the list of statements and removed any duplicate items and edited for spelling and grammar. The final list included 97 statements. In a subsequent activity, participants were asked to complete the sorting and rating steps during which they grouped similar the statements together and rated each statement on dimensions of feasibility and importance. The research team then conducted analysis and utilized multidimensional scaling (MDS) and cluster analysis to create a concept map of the statements.

### Instrument Development

#### Instrument Development (second level header) CSAT initial instrument development

After cluster analyses were completed, the grouped statements were reviewed by the study team and statements were selected to produce an initial measurement tool. Utilizing concept mapping and item development best practices, statements were selected from the concept mapping responses that best reflected the meaning of its domain. Statements were selected through a series of in person meetings by a multiprofessional team. This team included both implementation scientists (KP, DL, RH, SM) and clinicians (SM, JL). Each cluster was addressed individually and all items within the cluster were reviewed, along with their ratings. Disagreements in selection were resolved through discussion among the research team. Statements were edited upon selection into items usable on the measure. Editing included processes consistent with measurement development, such as removing double-barreled items. To help focus the assessment on the capacity for sustainability, each statement was edited so that CSAT respondents would be asked to assess the extent to which each existed in their clinic or setting on a Likert-type scale from 1 (no extent) to 7 (great extent). An example of editing was changing the statements “ensure ongoing champions exist” and “ongoing champions” to “The practice has engaged, ongoing champions”. Other examples can be seen in Additional File 4.

A total of seven statements were selected for each of the domains for a total of 49 indicators. Seven statements were selected at this stage to allow removal of poorly performing items and end up with five items per subscale. In our experience, this approach yields both better-performing items and subscales with clearer underlying conceptual constructs [13, 30].

#### Participants and recruitment

Data from two different participant samples were used for the development and testing of the CSAT instrument. First, a *pilot* sample (N = 120) was used for the initial development of the instrument, which included item selection, preliminary psychometrics testing, and usability testing. Second, a subsequent set of *early CSAT users* administered the final 35-item version of the CSAT. These data were used for more in-depth psychometric testing, including subdomain analyses and structural invariance testing (see below).

The first CSAT pilot sample of participants was selected from different clinical work environments as well as from different healthcare professions. Recruitment efforts used a snowball sampling approach and included identifying and contacting stakeholders that could potentially benefit from using the tool, as well as promoting the CSAT at local and national dissemination and implementation conferences. Respondents had the option to forward the link to peers or nominate individuals to complete the CSAT. To incentivize participation, all respondents were offered an optional tailored sustainability results report and the opportunity to enter a drawing for one of five $50 gift cards. The final pilot sample size was 120 participants (Table 1).

**Table 1:** CSAT development participant demographic characteristics

	Pilot (***N***=120)		Early users (***N***=166)	
Characteristic	N	%	N	%
*Profession*				
Nurse	18	16	42	27
Pharmacist	37	33	11	7
Physician	29	26	34	22
Admin/research	13	12	29	19
Ancillary	7	6	18	11
Other	8	7	22	14
*Position/role*				
Bedside provider	44	40	65	42
Unit level management	7	6	–	–
System leadership	6	5	60	38
Program leader	27	24	4	3
Other	27	24	27	17
*Environment*				
Academic medical center	67	60	65	42
Private practice	6	5	16	10
Community hospital	21	19	19	12
Community health center	6	5	48	31
Other	12	11	8	5
*Setting*				
Inpatient	56	55	35	25
Outpatient	26	26	79	56
Both	19	19	27	19
*Patient*				
Pediatric	54	53	49	32
Adult	47	47	94	63
Both	–	–	6	4

Note: Frequencies add up to less than sample totals because of missing responses

The second set of *early user* program participants (N = 166) came from two separate research studies. The first study recruited clinical staff working on the cancer control continuum in Missouri. This included primary care environments, screening programs, and cancer care centers that are focused on the diagnosis and treatment of those with cancer. The second study recruited participants in antimicrobial stewardship teams working to implement surgical prescribing guidelines. The contact at each site forwarded the CSAT to stakeholders they identified to participate. The early user sample size was 166 participants who represented a mix of professions and roles. The early users differed from the pilot sample with more early users representing adult care and outpatient settings (Table 1).

#### Data collection and instrument testing

Data collection was administered through an electronic survey on Qualtrics [31]. The assessment included the 49 CSAT items, as well as a small number of additional demographic questions, and tool usability questions. Demographic items included questions about the individual, their profession, and their practice setting. Usability questions were adapted from the System Usability Scale [32] and were used to understand participant reactions to completing the CSAT and give insight into any concerns with the functionality of the tool.

#### Data management and analyses

Data were downloaded, managed, and analyzed in R. A variety of descriptive, visualization, and psychometric analyses were conducted to explore the item, domain, and instrument characteristics of the CSAT, with particular emphasis on reliability. The *classical test theory* package (CTT, Version 2.3.3) in R was used to calculate measures of internal consistency, particularly Cronbach’s alpha.

Confirmatory factor analyses were done using the *lavaan* package (Version 0.6-5) [33]. Confirmatory factor analysis (CFA) is a powerful and appropriate tool for testing a hypothesized subscale structure in a measurement instrument [34, 35]. All CFA models were estimated using robust full-information maximum likelihood to efficiently handle any issues with normality and missing data [36]. Initially, CFA was applied to the pilot CSAT (with 7 items per subscale) to identify poorly performing items and test our hypothesized sustainability domain structure. Models were fit that allowed intercorrelations among latent constructs, which aligns with our conceptual approach that assumes sustainability conceptual domains are distinctive but interrelated. Poor items were those that had low variability and/or poor fit with the intended subscale. Once the final structure was determined, we reran the CFA on the smaller, final version of the CSAT (5 items per subscale) for the pilot, early adopter, and combined samples. For the CFA tests, we used three commonly used measures of model fit to assess model adequacy: comparative fit index (CFI), root mean square error of approximation (RMSEA), and the standardized root mean square residual (SRMR). These three fit indices were chosen due to their wide usage; they comprise both absolute (RMSEA, SRMR) and relative measures of fit (CFI), as well as including two measures that value parsimony (RMSEA, SRMR) [37]. Demographics were further assessed to understanding potential differences in responses and understanding how these variables might influence sustainability capacity. The Guidelines for Reporting Reliability and Agreement Studies was utilized for reporting (Additional File 2) [38].

## Results

### Concept mapping

**Fig. 1:**
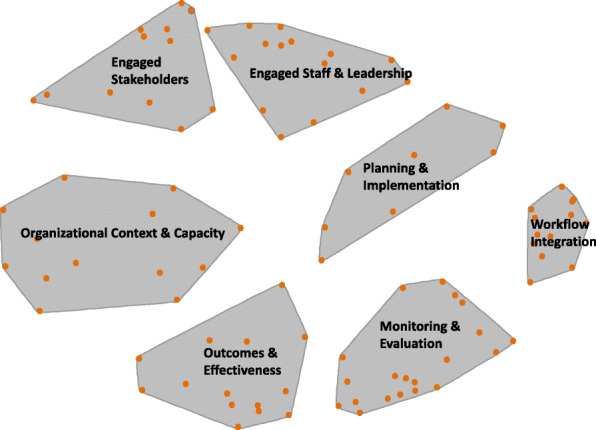
Seven conceptual domains of clinical sustainability, suggested by the concept mapping results

Figure 1 shows the concept mapping results after sorting the 97 statements. Seven domains emerged based on the sorts of the underlying items (points in the figure) and literature on sustainability. The resulting conceptual domains were *engaged staff and leadership*, *engaged stakeholders*, *monitoring and evaluation*, *organizational context and capacity*, *workflow integration*, *planning and implementation*, and *outcomes and effectiveness*. The domains represent determinants of sustainability capacity [39, 40]. These domains were then used to help develop the subscale structure of the clinical sustainability assessment tool.

**Fig. 2:**
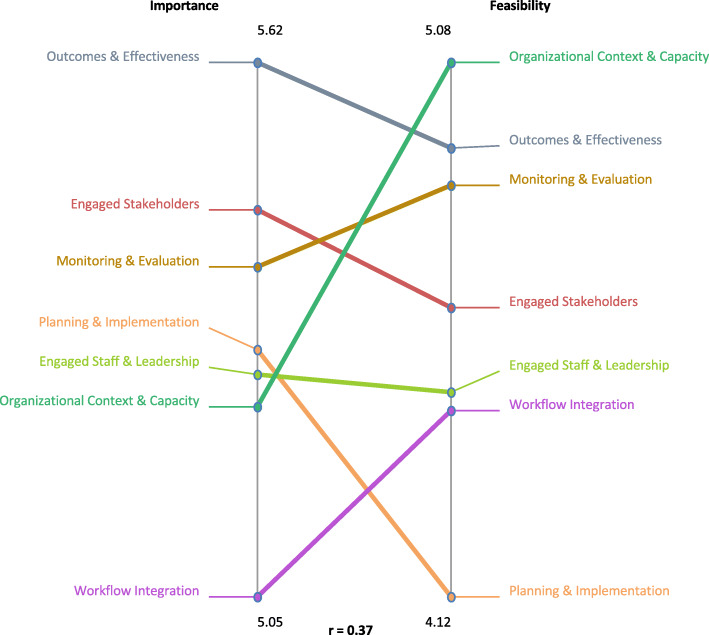
Pattern match map of importance and feasibility of seven domains of clinical sustainability

In order to understand more about the determinant domains, the mean cluster ratings of importance and feasibility were assessed through a pattern match map. Figure 2 shows the results of rating importance and feasibility. *Outcomes and effectiveness* was most highly rated for importance and *organizational context and capacity* was identified as most feasible. The overall correlation between importance and feasibility was r = 0.37.

### CSAT instrument development and pilot testing

#### Instrument improvement and domain structure

Item and confirmatory factor analyses were used to determine the final structure of the CSAT. Table 2 shows the improved psychometrics during the instrument development process. The baseline model is used as a comparison for the pilot, early adopter, and final models. The pilot original model includes all initial 49 items contained in seven subscales. After psychometric analyses, 14 items were dropped from the pilot original version of the framework and tool. Items were dropped if they had some combination of (1) lower loadings in the latent factors, (2) poor variance (i.e., restricted range), or (3) excessive missing data [41]. The final CSAT is comprised of 35 items organized within 7 subscale domains. Each domain represents a determinant of sustainability capacity and has five items. This simple and balanced structure facilitates training, scoring, and ease of use with practices and groups.

**Table 2:** Confirmatory factor analysis (CFA) results of baseline, pilot, early adopter, and final Clinical Sustainability Assessment Tool instruments

Phase	Subscales	Items	df	CFI	RMSEA	SRMR
Baseline comparison	1	49	1127	0.47	0.121	0.119
Pilot original	7	49	1106	0.68	0.095	0.094
Pilot final	7	35	539	0.81	0.084	0.075
Early users	7	35	539	0.91	0.063	0.052
All	7	35	539	0.93	0.051	0.049

Note: Total pilot N = 120; early adopters N = 166; all N = 286. CFA model fit with robust maximum-likelihood. *CFI* comparative fit index, *RMSEA* root mean square error of approximation, *SRMR* standardized root mean square residual

The final items of the CSAT within each of their subdomains can be seen in Additional file 1. The first domain, *engaged staff and leadership*, includes items assessing the extent to which the clinical practice has the support of internal frontline staff and management within the organization. *Engaged stakeholders* assesses the extent to which the practice has support among external stakeholders. *Organizational readiness* measures whether the organization has the internal supports and resources needed to effectively manage the practice. *Workflow integration* refers to whether the practice has been designed to fit into existing workplace processes, policies, and technologies (e.g., EMR systems). *Implementation and training* reflects whether the organization promotes processes and learning that appropriately guide the direction, goals, and strategies of the clinical practice. *Monitoring and evaluation* assesses the extent to which the organization monitors the clinical practice and uses data to inform quality improvement. Finally, *Outcomes and effectiveness* refers to whether and how the organization measures practice outcomes and impacts. Each subscale is scored separately (a simple average of the items in the subscale), and an overall CSAT score can be obtained, ranging from 1 to 7. Higher scores indicate a greater organizational capacity for clinical sustainability.

Table 2 also shows the very good fit of the 7-factor confirmatory factor analysis model to the data—that is, the 35 item CSAT does a credible job of measuring seven distinctive clinical sustainability domains that were identified in the literature and in the concept mapping phase of the study. The CFA results for the combined sample data (last row in Table 2) show an excellent fit of the model. Specifically, CFI scores of greater than 0.90 (here 0.93), RMSEA scores below 0.06 (here 0.051), and SRMR scores less than 0.08 (here 0.049) all are signs of excellent fit [42–45].

The most important pattern in the CFA results presented in Table 2 is the improvement in model fit as we move from a single factor model, to a seven-subscale model with all items, and finally, the seven-subscale model with the reduced number of items. The overall results suggest that the CSAT measures distinctive aspects of clinical sustainability with a relatively modest number of items that promote ease of use. Table 3 presents the intercorrelations among the seven latent constructs of the final CFA model, representing the interrelationships of these conceptual domains. Taken together, these results suggest that the seven constructs underlying the CSAT are distinctive, but interrelated. This is consistent with our conceptual framework which assumes that organizational capacity for sustainability is a multidimensional construct, made up of various aspects such as organizational readiness and workflow integration.

**Table 3:** Intercorrelations among seven CSAT subscale domains (latent constructs in CFA model)

	EStf	EStk	Org	Work	Imp	Mon	Out
Engaged staff and leadership							
Engaged stakeholders	.83						
Organizational readiness	.64	.64					
Workflow integration	.68	.69	.71				
Implementation and training	.73	.80	.75	.76			
Monitoring and evaluation	.73	.73	.67	.70	.83		
Outcomes and effectiveness	.71	.71	.59	.71	.74	.66	

#### Measurement invariance across samples

An important strength of this study is that we used two samples in the development and testing of the CSAT. The pilot sample was used to finalize the item selection and domain structure, while the two programs in the early users sample provided new data using the final CSAT instrument with 35 items. We conducted a measurement invariance test to determine if the CSAT domain structure varied across these two samples. Specifically, we used CFA to perform a configural invariance test [46], which tests whether the instrument subscales show similar patterns of item loadings across our two samples. Configural invariance means that the latent constructs have the same pattern loadings for each of the samples [47]. Our results were not significant (chi-squared difference test; χ^2^ = 30.03, *df* = 28, *p* = 0.36), indicating configural invariance and suggesting that the subdomain structure of the CSAT is the same across these multiple groups.

#### Subscale reliability

Table 4 presents the subscale reliabilities (internal consistency) for the CSAT for both samples. The average internal consistency of the seven subscales across both samples is 0.91, and range from 0.84 to 0.93. These indicate excellent scale reliability, especially given the small size of each subscale (5 items) [48]. Internal consistency tends to go up with more items, so a desirable goal is the fewest items that still maintain high reliability [42]. Furthermore, the item loadings show consistently high intercorrelations with their respective subscales, indicating a good fit of individual items with overall subscale scores (Additional File 3, full results available from authors).

**Table 4:** Subscale reliabilities (internal consistency) for the Clinical Sustainability Assessment Tool subscales

	Cronbach’s α		
Subscale	Pilot	Early users	All
Engaged staff and leadership	0.85	0.93	0.90
Engaged stakeholders	0.82	0.86	0.84
Organizational readiness	0.87	0.94	0.92
Workflow integration	0.89	0.93	0.92
Implementation and training	0.90	0.94	0.93
Monitoring and evaluation	0.94	0.93	0.93
Outcomes and effectiveness	0.90	0.94	0.93

Note: Each subscale is made up of 5 items

#### Preliminary CSAT results and validation

**Fig. 3:**
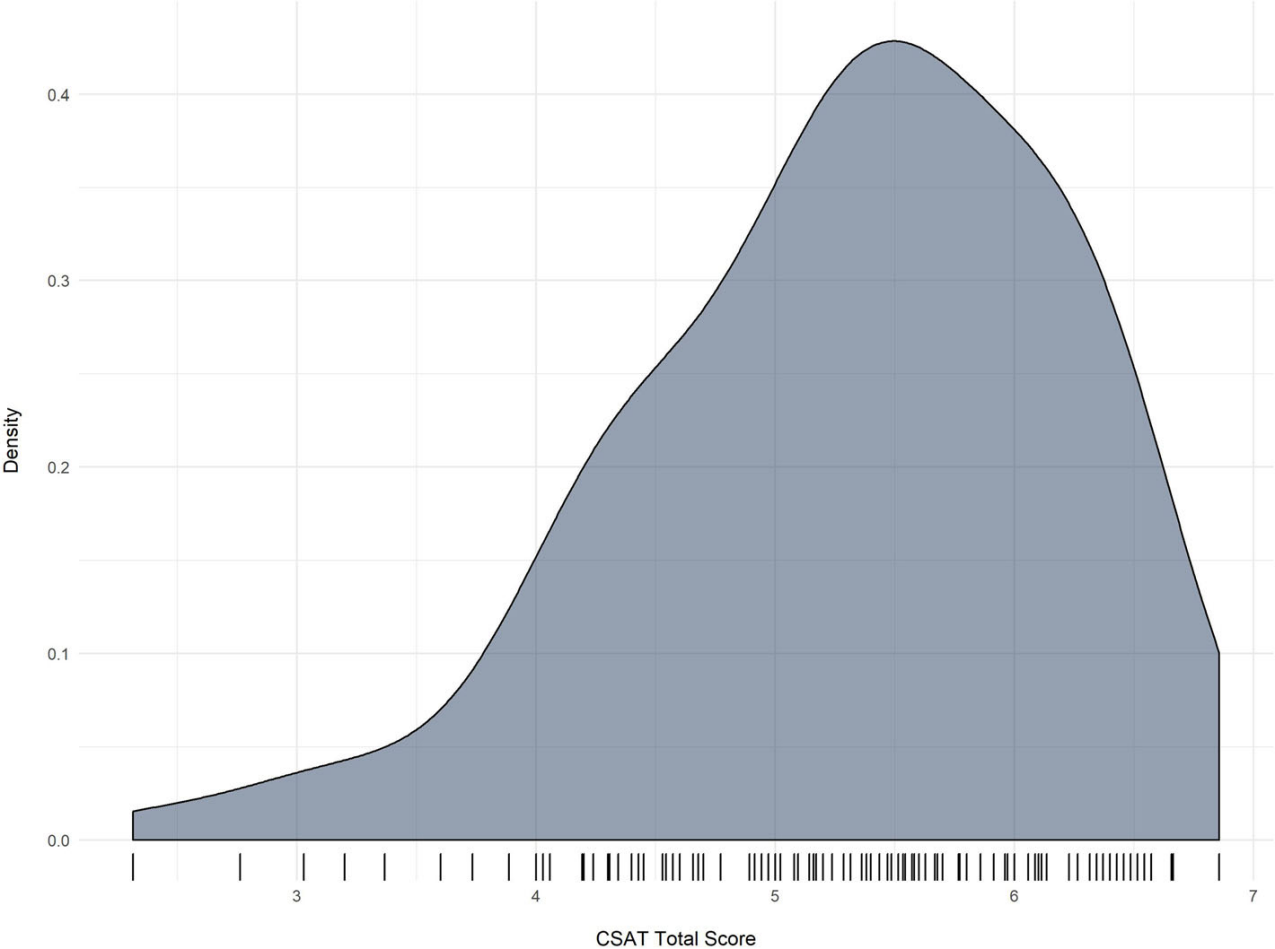
Variability of CSAT total scores

Subdomain scores are the simple means of the five items in each domain. The total CSAT score is then the mean of the seven subdomain scores. Table 5 presents descriptive statistics for the seven subscales and the total scores. *Organizational readiness* has the lowest average score, while *outcomes and effectiveness* is rated the highest. The standard deviations, ranges, and the density plot shown in Fig. 3 show that there is good variability of scores and no major problems with restricted range. The CSAT total scores range from 2.3 to 6.9. The standard deviation for the total scores is lower than for the subdomain scores, which is expected given that the total score is the mean of the seven subdomain scores.

**Table 5:** CSAT subscale and total score descriptive statistics (N = 120)

Subscale	Mean	SD	Low	High
Engaged staff and leadership	5.50	1.03	1.60	7.00
Engaged stakeholders	5.02	1.18	1.20	7.00
Organizational readiness	4.97	1.23	2.40	7.00
Workflow integration	5.49	1.13	1.60	7.00
Implementation and training	5.14	1.25	2.00	7.00
Monitoring and evaluation	5.12	1.41	1.00	7.00
Outcomes and effectiveness	5.95	1.01	1.60	7.00
Total score	5.29	0.90	2.31	6.86

In addition to the CSAT scores, basic characteristics of the clinical setting were collected (i.e., patient, setting, and environment type) as well as two characteristics of the respondent (i.e., job position, and training profession). As part of a set of preliminary validational analyses, CSAT total and subscale scores were examined across these five setting and respondent characteristics. In terms of setting characteristics, total CSAT scores varied significantly by setting type (*F* = 3.16, *p* = 0.047) and environment (*F* = 2.93, *p* = 0.038), but not by patient type (*F* = 1.09, *p* = 0.299). CSAT scores did not vary by respondent’s profession (*F* = 0.93, *p* = 0.449) or job position (*F* = 1.69, *p* = 0.175).

**Fig. 4:**
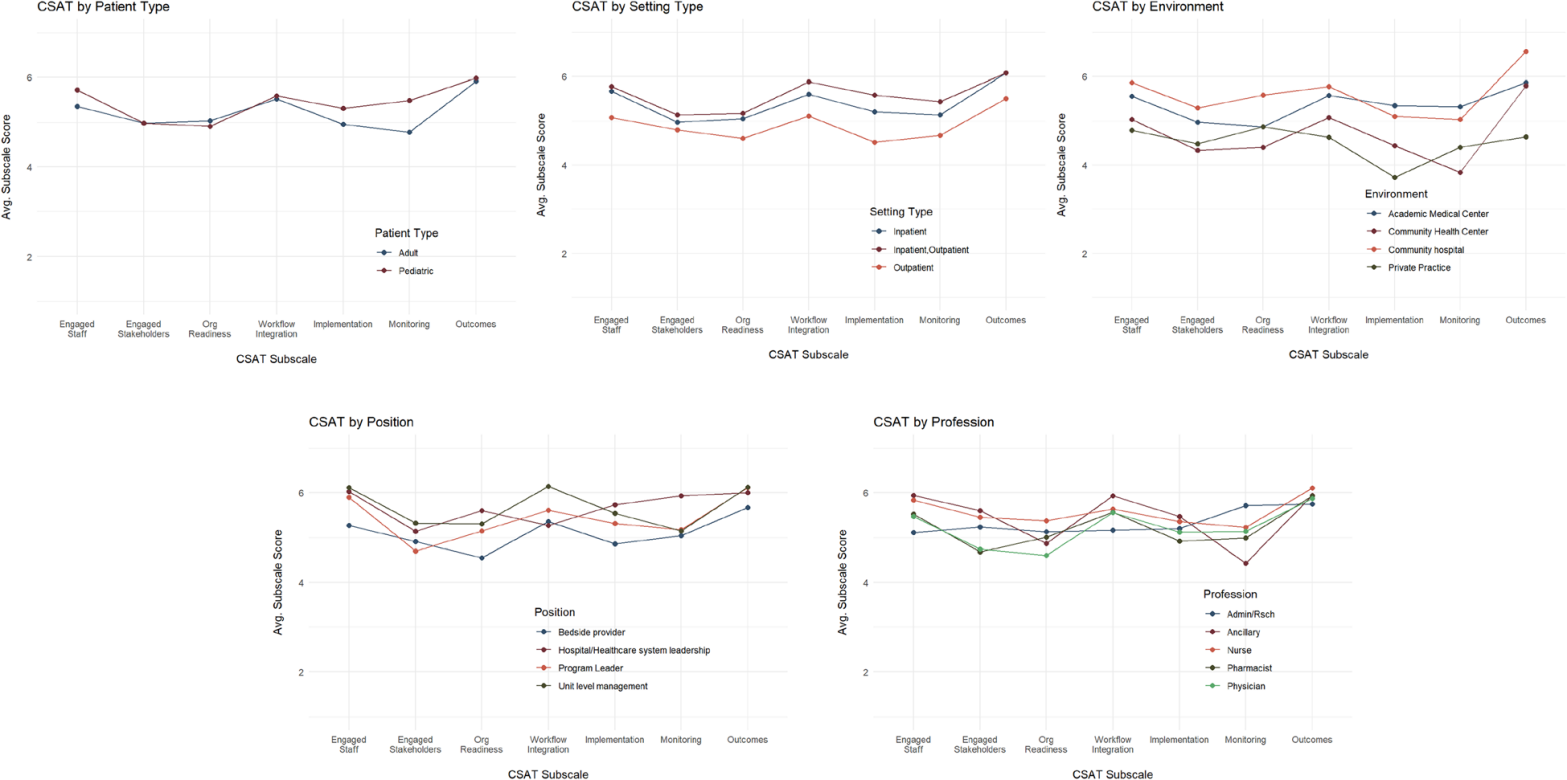
Profile plots for CSAT subscales by characteristics of the setting and respondents

Figure 4 shows the CSAT subscale score profile plots for the three setting (top row) and two respondent (bottom row) characteristics. Domain scores were very similar based on patient age category but showed significant differences based on outpatient vs. inpatient clinical setting and academic vs. community vs. private practice environment. Outpatient settings report consistently lower sustainability scores than inpatient settings across all seven domains. Academic medical centers and community hospitals were assessed as having higher sustainability capacities than community health centers and private practices. While the type of environment is a little more nuanced, it appears that community hospitals and academic medical centers report higher sustainability than community health centers and private practice settings.

#### Usability testing

We also collected data regarding ease of use and asked participants to report on their experience using the CSAT. On average, it took participants just under 20 min to complete the longer initial 49-item version of the CSAT and just under 10 min to complete the final 35-item tool. Participants also rated the experience of completing the CSAT in a positive manner: 85% of participants rated the tool as easy to use; 75% felt very confident about their ability to use the tool; 90% thought that most other people would be able to learn quickly how to use the tool. Importantly, only 35% thought that they would need external support to use the tool effectively.

In addition to the usability data from our pilot participants, we assessed the final 35-item version of the CSAT using the Psychometric and Pragmatic Evidence Rating Scale (PAPERS), which offers an objective rating of measure quality [49]. The PAPERS pragmatic scale scores five practical characteristics of measures on a Likert scale ranging from -1 (poor) to 4 (excellent) for a maximum total score of 20 indicating the highest pragmatic quality. The CSAT rates 3 (good) or 4 (excellent) in each of the five PAPERS pragmatic categories: brevity, cost, ease of training, ease of interpretation, and language. The CSAT rates good in brevity with fewer than 50 items and language at less than a 10th grade reading level. It rates excellent in cost, ease of training, and ease of interpretation being free, requiring no training to use, and automated calculations of scores. The total PAPERS pragmatic score is 18/20, indicating this is a highly practical, usable instrument.

## Discussion

We introduce here the Clinical Sustainability Assessment Tool (CSAT) as a reliable instrument to assess organizational capacity for sustainability in clinical settings. The final CSAT includes seven distinct determinant domains with high internal consistency within each domain. The CSAT also demonstrates good variability with respect to clinical setting and practice, but maintains consistency across respondents and patients.

More specifically, these results provide initial discriminant validational support for the CSAT. The CSAT is designed to assess determinants of sustainability for specific clinical practices. These settings are often associated with different characteristics, both related to practice adaptation and environmental context. Because sustainability requires a team effort, the assessment of sustainability should not be different based on the training and/or job title of the people providing the assessment. Here, with the small pilot sample, we can see that the CSAT is able to detect perceived sustainability differences among different types of clinical settings and environments.

This instrument is of potential value to researchers in implementation science, as well as evaluators of clinical practices and outcomes. Our measure aligns with existing frameworks conceptualizing organizational capacity and emphasizes specific concepts known to be relevant to clinical settings [9, 50]. For example, workflow is known to have a significant impact on the implementation of new interventions in clinical settings [51]. Similarly, we would hypothesize that new interventions that are poorly integrated into the existing clinical workflow will be less likely to be sustained.

Other instruments exist that assess organizational context and have been well-validated and widely used: Organizational Readiness for Change Assessment (ORCA), Alberta Context Tool, and the Implementation Leadership Scale are frequently used tools [52–54]. The CSAT adds to this toolbox in important ways. Unlike the ORCA, which focuses on initial implementation, the CSAT specifically focuses on sustainability but can be used in all phases of implementation. Indeed, planning for sustainability in the early implementation phases improves likelihood of long-term sustainment [4]. In addition, unlike the Alberta Context Tool and the Implementation Leadership Scale, which assess general aspects of the organization, the CSAT focuses on sustaining a specific intervention rather than a general measurement of context. This allows users to focus their sustainability assessment on specific interventions. The CSAT’s ability to identify concrete interventions and activities will allow researchers to understand aspects of capacity that are essential to sustaining specific interventions, to recognize patterns across different types of clinical settings attempting to sustain the same intervention, and to describe the impact of organizational capacity on different kinds of interventions [55].

Our instrument also pairs well with the goals of health systems and quality improvement research. It aims to assist with sustainment of an identified clinical practice, which would support overall quality care. Further, this applies across the context of specific practice improvement, a traditional focus of quality improvement work, while also focusing on broader contextual and process factors that are highlighted in implementation science work [56–58].

We intended this tool not only to be reliable and valid, but also to be brief and easy to use in busy clinical settings, making the CSAT of high value to clinicians. To allow equal and open access, the CSAT and the companion assessment for public health settings, the PSAT, are both freely available. Online, clinicians may fill out a version of the tool and receive a report summarizing their CSAT score and recommendations for improving organizational capacity for sustaining clinical interventions. This simple structure, which may be used by one individual or a group, facilitates training and implementation planning. The complete CSAT instrument, along with instructions, is also available in Additional file 1.

Future CSAT research and evaluation work is planned to enhance our knowledge of how best to measure capacity for sustainability in clinical settings, to determine how it operates within broader implementation studies, and to assess its predictive validity for implementation outcomes in healthcare systems and clinical settings. Collectively, this work will address the major limitation of this study, which is the relatively small convenience sample. We are also exploring other ways to enhance its use for broader audiences, including the development of a brief CSAT version, and translating the CSAT into other languages, starting with Spanish.

## Conclusion

The full benefit of evidence-based interventions will continue to go unrealized without sustained delivery of interventions over time. A critical precursor to sustaining intervention is having adequate organizational capacity. To date, researchers and practitioners have lacked adequate measures to assess organizational capacity for sustainability. The CSAT is a valid, reliable, and easy to use tool that may be used to assess capacity, support sustainability research, and promote sustainable healthcare service delivery.

## Supplementary Information

**Additional file 1.** CSAT

**Additional file 2.** Reporting checklist

**Additional file 3.** CFA detailed results

**Additional file 4.** Statements to items

## Data Availability

The datasets generated and/or analyzed during the current study are not publicly available due to their containing information which could compromise the confidentiality, but are available from the corresponding author on reasonable request.

## Abbreviations

PSAT: Program Sustainability Assessment Tool
NHS: National Health Service
CSAT: Clinical Sustainability Assessment Tool
CFI: Comparative fit index
RMSEA: Root mean square error of approximation
SRMR: Standardized root mean square residual
CFA: Confirmatory factor analysis
ORCA: Organizational Readiness to Change Assessment
PAPERS: Psychometric and Pragmatic Evidence Rating Scale
