# Supplementary material for: The Clinical Sustainability Assessment Tool: measuring organizational capacity to promote sustainability in healthcare
Source: Implement Sci Commun. 2021 Jul 17;2:77. doi: 10.1186/s43058-021-00181-2 (PMC8285819; doi:10.1186/s43058-021-00181-2)
Supplement: Supplementary file 2 — Additional file 2. Reporting checklist [file 43058_2021_181_MOESM2_ESM.docx]

Title and Abstract:

1. Identify in title or abstract that interrater/intrarater reliability or agreement was investigated
   1. This was not the type of reliability we assessed, but our abstract outlines the reliability assessment completed

Introduction:

1. Name and describe the diagnostic or measurement device of interest explicitly
   1. The Clinical Sustainability Assessment Tool –the description of the tool creation and pilot testing is included
2. Specify the subject population of interest
   1. The area of interest is specified.
3. Specify the rater population of interest (if applicable)
   1. Not applicable
4. Describe what is already known about reliability and agreement and provide a rationale for the study (if applicable)
   1. New tool creation and pilot – none known about this measure

Methods:

1. Explain how the sample size was chosen. State the determined number of raters, subjects/objects, and replicate observations
   1. The sample is described for the pilot testing, the data utilized in analysis
2. Describe the sampling method
   1. It is described in the methods section
3. Describe the measurement/rating process (e.g., time interval between repeated measurements, availability of clinical information, blinding)
   1. Our process is described in the methods section.
4. State whether measurements/ratings were conducted independently
   1. The individuals completed the assessment individually (described).
5. Describe the statistical analysis.
   1. Described under *Data Management & Analyses*

Results:

1. State the actual number of raters and subjects/objects that were included and the number of replicate observations that were conducted
   1. No replicate observations, number of subjects and demographics is presented.
2. Describe the sample characteristics of raters and subjects (e.g., training, experience)
   1. Appropriate characteristics are described
3. Report estimates of reliability and agreement including measures of statistical uncertainty
   1. Appropriate statistical analyses reported.

Discussion:

1. Discuss the practical relevance of results.
   1. Completed in the discussion section

Auxiliary Material:

1. Provide detailed results if possible (e.g., online)
   1. Appropriate information is provided.

From:

Kottner J, Audige L, Brorson S, Donner A, Gajewski BJ, Hróbjartsson A et al. Guidelines for Reporting Reliability and Agreement Studies (GRRAS) were proposed. International Journal of Nursing Studies. 2011;48(6):661-71. doi:<https://doi.org/10.1016/j.ijnurstu.2011.01.016>.
