# Supplementary material for: The Clinical Sustainability Assessment Tool: measuring organizational capacity to promote sustainability in healthcare
Source: Implement Sci Commun. 2021 Jul 17;2:77. doi: 10.1186/s43058-021-00181-2 (PMC8285819; doi:10.1186/s43058-021-00181-2)
Supplement: Supplementary file 3 — Additional file 3. CFA detailed results [file 43058_2021_181_MOESM3_ESM.docx]

Additional File 3 – Detailed CFA analysis results and diagnostics for final seven domain CSAT model.

Figure AF3-1. Seven domain CSAT measurement model, including standardized indicator residuals and factor loadings. Note: EStf = Engaged Staff & Leadership; EStk = Engaged Stakeholders; Org = Organizational Readiness; Wr = Workflow Integration; Imp = Implementation & Training; Mon = Monitoring & Evaluation; Out = Outcomes & Effectiveness.


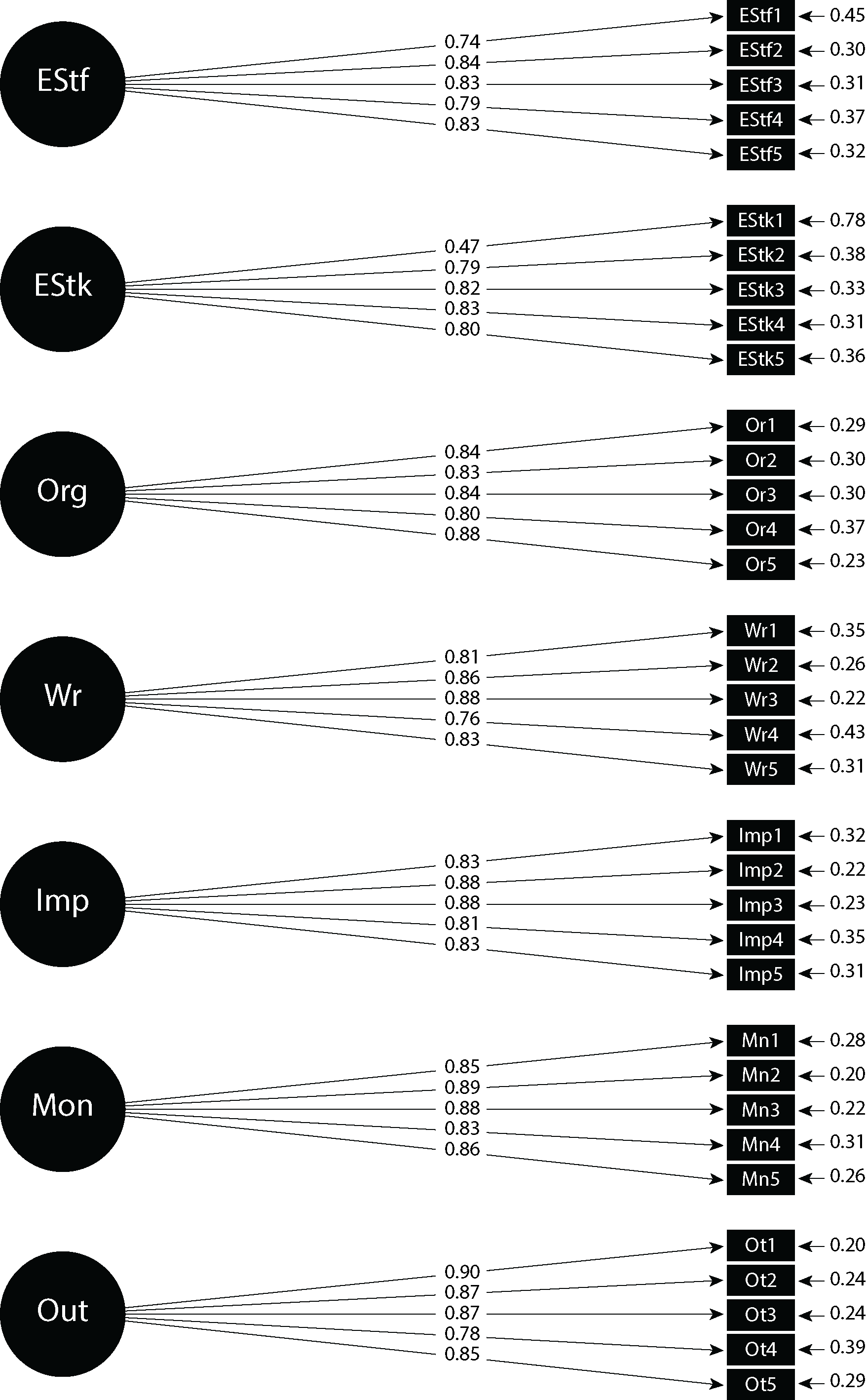


Figure AF3-2. Density plot of final CSAT CFA model standardized residuals (correlations). The small sizes of the correlations and the normal distribution both indicate a well-fitting model.


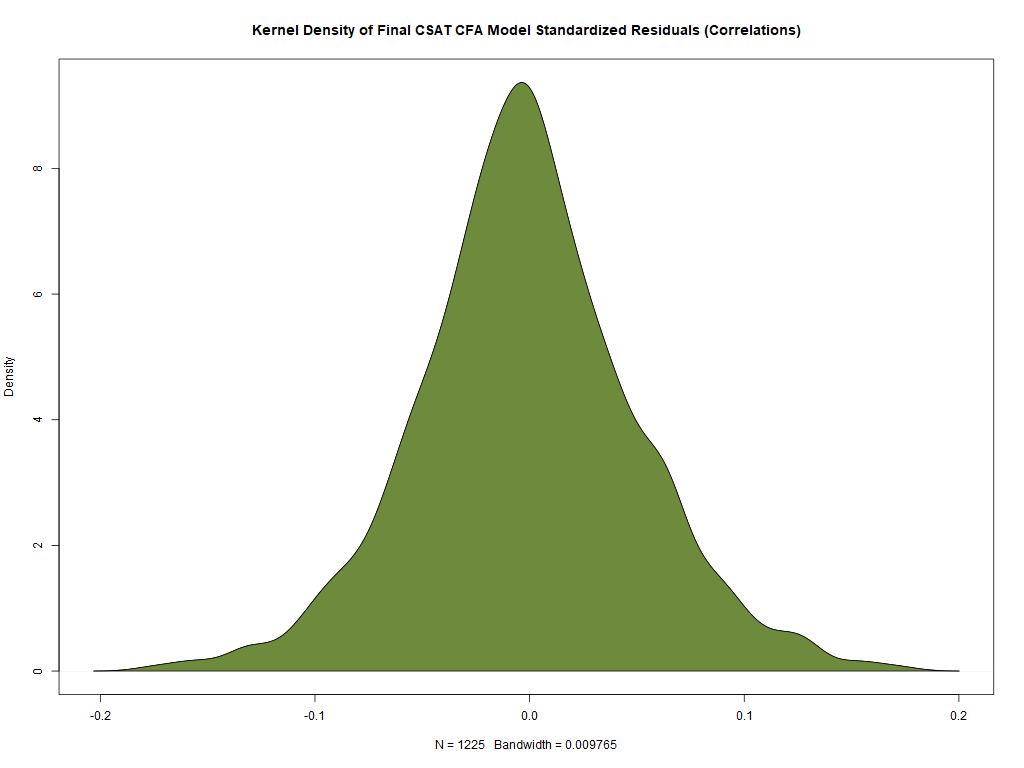


Table AF3-1. Intercorrelations among seven CSAT subscale domains (latent constructs in CFA model).

|  | EStf | EStk | Org | Work | Imp | Mon | Out |
| --- | --- | --- | --- | --- | --- | --- | --- |
| Engaged Staff & Leadership |  |  |  |  |  |  |  |
| Engaged Stakeholders | .83 |  |  |  |  |  |  |
| Organizational Readiness | .64 | .64 |  |  |  |  |  |
| Workflow Integration | .68 | .69 | .71 |  |  |  |  |
| Implementation & Training | .73 | .80 | .75 | .76 |  |  |  |
| Monitoring & Evaluation | .73 | .73 | .67 | .70 | .83 |  |  |
| Outcomes & Effectiveness | .71 | .71 | .59 | .71 | .74 | .66 |  |

Table AF3-2. Ten largest modification indices for final CSAT CFA model, taken from *lavaan* output. The table uses *lavaan* operator syntax, where ‘~~’ designates a covariance between two indicators (observed variables), and `=~` designates an indicator loading on a latent construct. For the final CSAT model, we see that if we allowed a covariance between Org3 and Org4, then the χ^2^ for the overall model fit would improve by 90.2. This is actually not very large, given that the χ^2^ for the final model is 1273. In any case, we do not wish to improve the model by following any of these modification index suggestions—that would risk overfitting the model and would not align with how the CSAT would be used and scored by researchers and evaluators in the ‘real world’.

| *Index #* | *LHS* | *Operator* | *RHS* | *MI Value* |
| --- | --- | --- | --- | --- |
| 1 | Org3 | ~~ | Org4 | 90.2 |
| 2 | EStf4 | ~~ | EStf5 | 35.1 |
| 3 | EStf2 | ~~ | EStf3 | 34.2 |
| 4 | Org3 | ~~ | Work5 | 19.2 |
| 5 | EStf5 | ~~ | Plan1 | 16.1 |
| 6 | Work | =~ | Mon1 | 16.1 |
| 7 | EStf5 | ~~ | Plan4 | 16.0 |
| 8 | Work3 | ~~ | Mon5 | 16.0 |
| 9 | Out | =~ | Org3 | 15.9 |
| 10 | Out | =~ | Mon12 | 15.7 |

Note: Complete analytic results for all tested models are available from the authors.
