# Supplementary material for: The Clinical Sustainability Assessment Tool: measuring organizational capacity to promote sustainability in healthcare
Source: Implement Sci Commun. 2021 Jul 17;2:77. doi: 10.1186/s43058-021-00181-2 (PMC8285819; doi:10.1186/s43058-021-00181-2)
Supplement: Supplementary file 4 — Additional file 4. Statements to items [file 43058_2021_181_MOESM4_ESM.docx]

| **Domain** | **Brainstorm statements** | **Tool items** |
| --- | --- | --- |
| Engaged staff & leadership | ·       Ensure ongoing champions exist  ·       Engaged champions | The practice has engaged, ongoing champions. |
| Implementation & training | ·       Consistent education across professions  ·       Ongoing education for new team members and to refresh established team members. | The practice has ongoing education across professions. |
| Engaged Stakeholders | ·       Respect for all stakeholders | There is respect for all stakeholders involved in the practice. |
| Workflow integration | ·       Consistency in practice | The practice is designed to be used consistently. |
| Monitoring & Evaluation | ·       To be monitored and reported on  ·       Review on a consistent basis | Evaluation and monitoring of the practice are reviewed on a consistent basis. |
| Outcomes & Effectiveness | ·       To demonstrate clear relative advantage over alternatives | The practice has clear advantages over alternatives. |
| Organizational Readiness | ·       To integrate well with established clinical practices.  ·       To be incorporated in existing processes | The practice is well integrated into the operations of the organization. |
